# Development and validation of a clinical index to predict survival after cardiac resynchronisation therapy

**DOI:** 10.1136/hrt.2009.173880

**Published:** 2009-07-09

**Authors:** F Leyva, P W X Foley, B Stegemann, J A Ward, L L Ng, M P Frenneaux, F Regoli, R E A Smith, A Auricchio

**Affiliations:** 1 University of Birmingham, Department of Cardiology, Good Hope Hospital, Heart of England NHS Trust, Sutton Coldfield, UK; 2 Medtronic Inc, Bakken Research Center, Maastricht, The Netherlands; 3 University of Birmingham, Birmingham, UK; 4 University of Leicester, Leicester, UK; 5 Queen Elizabeth Hospital, University of Birmingham, Birmingham, UK; 6 Fondazione Cardiocentro Ticino, Lugano, Switzerland

## Abstract

**Objective:**

To develop and validate a prognostic risk index of cardiovascular mortality after cardiac resynchronisation therapy (CRT).

**Design:**

Prospective cohort study.

**Setting:**

District general hospital.

**Patients:**

148 patients with heart failure (mean age 66.7 (SD 10.4) years), New York Heart Association class III or IV, LVEF <35%) who underwent CRT.

**Interventions:**

CRT device implantation.

**Main outcome measures:**

Value of a composite index in predicting cardiovascular mortality, validated internally by bootstrapping. The predictive value of the index was compared to factors that are known to predict mortality in patients with heart failure.

**Results:**

All patients underwent assessment of 16 prognostic risk factors, including cardiovascular magnetic resonance (CMR) measures of myocardial scarring (gadolinium-hyperenhancement) and dyssynchrony, before implantation. Clinical events were assessed after a median follow-up of 913 (interquartile range 967) days. At follow-up, 37/148 (25%) of patients died from cardiovascular causes. In Cox proportional hazards analyses, (DSC) Dyssynchrony, posterolateral Scar location (both p<0.0001) and Creatinine (p = 0.0046) emerged as independent predictors of cardiovascular mortality. The DSC index, derived from these variables combined, emerged as a powerful predictor of cardiovascular mortality. Compared to patients with a DSC <3, cardiovascular mortality in patients in the intermediate DSC index (3–5; HR: 11.1 (95% confidence interval (CI) 3.00 to 41.1), p = 0.0003) and high DSC index (⩾5; HR: 30.5 (95% CI 9.15 to 101.8), p<0.0001) were higher. Bootstrap validation confirmed excellent calibration and internal validity of the prediction model.

**Conclusion:**

The DSC index, derived from a standard CMR scan and plasma creatinine before implantation, is a powerful predictor of cardiovascular mortality after CRT.

The benefits of cardiac resynchronisation therapy (CRT) are well established. The Cardiac Resynchronisation in Heart Failure (CARE-HF) study showed that, compared to optimum medical therapy, CRT-pacing (CRT-P) led to a 36% relative reduction in total mortality.^1^ The Comparison of Medical Therapy, Pacing and Defibrillation in Heart Failure (COMPANION) study showed that addition of a cardioverter defibrillator (CRT-D) led to higher survival benefits than CRT-P.^2^ Predicting which patients derive a survival benefit from CRT, however, remains a challenge. Numerous echocardiographic studies have focused on measures of cardiac dyssynchrony as predictors of outcome,^3^ but no consensus has been reached as to their clinical applicability. In the recent Predictors of Response to CRT (PROSPECT) study, which adopted left ventricular (LV) reverse remodelling as a surrogate of survival, tissue Doppler imaging-based measures of dyssynchrony were proved to add little value to QRS duration in predicting the outcome of CRT.^4^ No echocardiographic measure has been validated against mortality after CRT.

Cardiovascular magnetic resonance (CMR) is the gold standard for the in vivo assessment of myocardial structure, function and dyssynchrony.^5^ A recent pilot study has shown that a dyssynchrony measure derived from a standard CMR scan, the CMR tissue synchronisation index (CMR-TSI), is a powerful predictor of mortality and morbidity after CRT.^6^ As well as providing measures of dyssynchrony, CMR also allows visualisation and quantification of myocardial scarring with late gadolinium enhacement (CMR-LGE).^7^ ^8^ Importantly, the size (burden), transmurality and location of a myocardial scar in the LV free wall, the target location for LV leads, have been linked to a poor outcome after CRT.^7^ ^8^ ^9^

We have explored the value of pre-implant CMR measures of dyssynchrony and myocardial scarring, as well as more conventional heart failure risk factors,^10^ in predicting cardiovascular mortality after CRT. This study focuses on the derivation and validation of a clinical risk index based on a combination of such non-invasive measures.

## Methods

### Patients

A total of 148 consecutive heart failure patients were prospectively enrolled from January 2001 to April 2008 at a single centre (Good Hope Hospital). Patients were selected if they were due to undergo CRT for standard indications (ischaemic or non-ischaemic cardiomyopathy, New York Heart Association (NYHA) class III or IV, LV ejection fraction (LVEF) <35%, QRS duration ⩾120 ms). Until publication of the UK National Institute for Health and Clinical Excellence (NICE) Health Technology Appraisal in CRT in 2007,^11^ the implantation rate for CRT-D in the UK was very low. All patients included in this study underwent CRT-P.

All patients had undergone coronary angiography. The clinical diagnosis of systolic heart failure was based on clinical criteria and evidence of systolic dysfunction on echocardiography. The diagnosis of ischaemic cardiomyopathy was made if systolic dysfunction was associated with a history of myocardial infarction,^12^ the findings of coronary angiography, or if the pattern of LGE was characteristic of a myocardial infarction.^13^ All patients gave written informed consent and the study was approved by the North Birmingham ethics committee.

### Study design

A CMR study was performed at baseline. The predictive risk index was validated against cardiovascular death. For qualitative comparisons with the CARE-HF study, we also considered the composite endpoint of death from any cause or hospitalisations for major cardiovascular events, as defined in the CARE-HF study protocol.^1^ Mortality data were collected through medical records and, where appropriate, from interviews with patients’ caregivers. Clinical outcome data were collected on a two-monthly basis by an investigator who was blinded to other clinical and imaging data. Sudden cardiac death was defined as “a natural, unexpected death due to cardiac causes, heralded by an abrupt loss of consciousness within one hour of the onset of acute symptoms or occurring during the sleep without worsening of heart failure in the preceding hours.”^14^

### Device therapy

Implantation of CRT-P devices was undertaken using standard techniques under local anaesthesia. The LV lead position was chosen by the implanter, who was blinded to the CMR study. Patients who underwent successful implantation were followed up in a dedicated device therapy clinic. Patients in sinus rhythm underwent trans-mitral Doppler-directed optimisation of atrioventricular delay^15^ before discharge and at every scheduled visit thereafter. For patients in atrial fibrillation, right ventricular and LV leads were implanted and a biventricular generator was used, plugging the atrial port and programming the generator to a ventricular-triggered mode. All patients were proved to have functional leads and generators throughout the study period.

### Cardiovascular magnetic resonance

Imaging was performed using a 1.5 Tesla scanner (Signa, GE Healthcare, Slough, UK) and a phased array cardiac coil. A short axis LV stack was acquired using a steady state in free precession sequence (SSFP, repetition time 3.0–3.8 ms; excitation time 1.0 ms; image matrix 224×224; field of view 36–42 cm; flip angle 45°) in sequential 8-mm slices (2-mm interslice gap) from the atrio-ventricular ring to apex. Acquisition was performed during gated 8-second breath-holds. Left ventricular end diastolic volume (LVEDV) and end-systolic (LVESV) volume, and LVEF were quantified using manual planimetry of all short-axis SSFP cine images with MASS analysis software (Medis, Leiden, The Netherlands). The observer was blinded to echocardiographic and clinical data.

The CMR-TSI was measured as previously described.^6^ Briefly, the maximum radial wall motion value of a radial wall motion time series was chosen as the peak radial wall motion for each LV segment. The time-dependent segmental radial wall motion data (y) were fitted to an empirical sine wave function y  =  a + b × sin (t/RR + c). The mean segmental radial wall motion (a), the segmental radial wall motion amplitude (b) and the segmental phase shift of the maximum radial wall motion (c) were extracted from the fit. The non-linear least squares fit function implemented by Bates and DebRoy into the “nls” packet of R was used for the sine fit.^16^ The CMR-TSI was expressed as the SD of all segmental phase shifts of the radial wall motion extracted from the fit. Therefore, the CMR-TSI is a measure of the temporal dispersion of peak inward myocardial motion throughout the cardiac cycle. A high CMR-TSI, therefore, denotes high temporal dispersion of inward wall motion.

For scar imaging using CMR-LGE, short-axis slices identical to the LV stack were acquired using a segmented inversion-recovery technique 10 minutes after the intravenous administration of gadolinium-diethylenetriamine pentaacetic acid (0.1 mmol/kg). Inversion times were adjusted to null normal myocardium (260–400 ms). Scar volume was calculated by multiplying the planimetered area of LGE in each slice by the slice thickness. Scar volume was expressed as a percentage of LV myocardial volume in the diastolic phase. Areas of scar were then segmented using a 17-segment model.^17^ A posterolateral scar was defined as a scar which, in this model, was located in segments 6, 12, 16, 5 or 11, or in the lateral half of segments 1, 7, 13, 17, 15, 10 and 4. Transmurality was also assessed using a 17-segment model.^17^ A transmural posterolateral scar was defined as a scar with 51–100% of the LV wall, measured radially.

### Statistical analysis

Continuous variables are expressed as mean (SD). Normality was tested using the Shapiro-Wilk test. Variables which were not normally distributed were log-transformed before statistical analyses. Comparisons between normally distributed continuous variables were made using ANOVA with Scheffe’s F procedure for multiple comparisons. Categorical variables were analysed using χ^2^ tests and Scheffe’s post-hoc test. Differences in Kaplan-Meier survival curves between the risk groups were assessed using the log-rank (Mantel-Cox) test. Areas under the receiver-operating characteristic (ROC) curves (AUC) were used to derive optimal cut-off points for the DSC index. Statistical analyses were performed using Statview and SPSS 13.0. Harrel’s “Design” package version 2.1-1in R was used for predictive modelling.^18^ A two-tailed p value of <0.05 was considered statistically significant. In a previous study,^6^ intra-observer and inter-observer variabilities for CMR-TSI were 3.0% and 8.8%, respectively.

#### Development of the risk index

In line with Harrell’s recommendations on multivariate prognostic modelling,^19^ ^20^ no more than *m*/10 parameters were considered, where *m* is the number of uncensored events, in this case cardiovascular deaths (n = 37). Candidate variables were selected on the basis of their proved clinical, pathophysiological and epidemiological relevance to the endpoint in question. A measure of cardiac dyssynchrony—namely, CMT-TSI, was included in view of previous demonstration from echocardiographic^21^ and radionuclide^22^ studies that extremes of dyssynchrony relate to a poor outcome in patients with heart failure. Similarly, a posterolateral location of myocardial scar was chosen in view of its negative influence on outcome after CRT.^8^ ^9^ Plasma creatinine was also selected given its emergence as a predictor of mortality in patients in heart failure in numerous studies.^10^ ^23^ ^24^ ^25^ ^26^ ^27^ These three variables were included in the multiple regression model. Continuous rather than dichotomous variables were used wherever possible.^20^ Interactions between variables were extensively explored using interaction terms in Cox proportional hazards models. Linearity assumptions were checked using Martingale residuals. Cross-product terms were added to check for additivity assumptions. The proportional hazards assumption was checked using Schoenfeld residuals.^28^

#### Validation

Validation was implemented using bootstrapping.^19^ ^20^ ^29^ This non-parametric method, which estimates sampling distributions of a predictor variable by resampling with replacement from the original sample, is, in effect, an impression of the validity of predictions in new but similar patients. For each group of 200 bootstrap samples, the model was refitted and tested against the observed sample in order to derive an estimate of the predictive accuracy and bias. This was used to select 148 “new” patients from the original cohort, one at a time with replacement, 200 times over. For each of these 200 samples, the following parameters were derived: (1) the β coefficients from Cox proportional hazards models; (2) Somer’s D rank correlation index and (3) an estimate of the slope shrinkage.^19^ The apparent Somer’s D (D_app_) was derived using stepwise Cox proportional analyses. The bootstrap-corrected performance of the predictor equation, or “optimism”,^20^ was quantified by assessing the difference between Somer’s D in the original sample (D_orig_) and D in the bootstrap sample (D_boot_). The average optimism, termed O, was derived by repeating the above steps 200 times over. The bootstrap-corrected performance of the original stepwise model, D_app_ – O, is, effectively, an honest estimate of internal validity.^20^ Actual survival was plotted against apparent estimated survival and bootstrap-corrected estimated survival.

#### Calculation of the risk index

The risk prediction index was expressed as the absolute value of the sum of the products of these variables and their β coefficients (that is, weights) from the final stepwise multivariate Cox proportional hazards model, identified by Harrels’s routine^19^ ^20^: Σ β_i_*x*_i_  =  |β_1_*x*_1_+β_2_*x*_2_+…+β_n_*x*_n_|, where *x*_1_, *x*_2_,…*x*_n_ are the values for the variables and β_1_, β_2_,… β_n_ are the coefficients for each variable. For categorical variables, presence was computed as the β coefficient and absence as zero.

#### Univariate analyses

Univariate Cox proportional hazards analyses were performed after development and validation of the DSC index. These were undertaken in order to assess the relative strength of other variables in relation to cardiovascular mortality.

## Results

The characteristics of the study group are shown in table 1. There were 20 cardiovascular deaths by one year and 28 by two years. After a follow-up of 913 (967) days (median (interquartile range) for events, there were 37 cardiovascular deaths. Of these, 28 were due to pump failure, eight were sudden and one patient underwent cardiac transplantation. Of the 48 patients with a posterolateral scar, 35 had a transmural scar and 12 had a non-transmural scar. Scar volume in these 48 patients was 33.2% (18.5).

**Table 1:** Clinical characteristics of the study group

Characteristics	n = 148
Age (years)	66.7 (10.4)
Men (% of patients)	114 (77)
Ischaemic aetiology (% of patients)	92 (62)
NYHA class	3.2 (0.43)
Creatinine (μmol/l)	119.8 (38.3)
Sodium (mmol/l)	139.0 (5.8)
Haemoglobin (g/dl)	14.1 (7.9)
**Co-morbidity, No (% of patients)**	
Diabetes mellitus	24 (16)
Hypertension	40 (27)
CABG	31 (21)
**Medication, No (% of patients)**	
Loop diuretics	129 (87)
ACE-I or ARB	136 (92)
β-blockers	81 (55)
Spironolactone	66 (45)
**ECG variables**	
Atrial fibrillation, No (% of patients)	23 (16)
QRS duration (ms)	145.9 (26.5)
**CMR variables**	
LVEDV (ml)	247.8 (99.5)
LVESV (ml)	200.2 (96.6)
LVEF (%)	22.5 (10.6)
CMR-TSI (ms)	100.7 (47.8)
Scar*	
Volume (% of LV myocardial volume)	19.5 (20.1)
Transmural, No (% of patients)	62 (42)
Posterolateral, No (% of patients)	48 (32)

Continuous variables are expressed as mean (SD).

*Transmural scars were those with ⩾51% transmurality.

ACE-I, angiotensin-converting enzyme inhibitors; ARB, angiotensin-receptor blockers; CABG, coronary artery bypass graft; CMR-TSI, cardiovascular magnetic resonance tissue synchronisation index; LVEDV, left ventricular end-diastolic volume; LVEF, left ventricular ejection fraction; LVESV, left ventricular end-systolic volume; NYHA, New York Heart Association.

### Derivation and validation

Table 2 shows model outputs for the variables included in the prediction model (DSC): Dyssynchrony, Scar location (posterolateral) and Creatinine. The DSC index was calculated as follows: (2.5039 if posterolateral scar present; 0 if absent) + 0.0107. CMR-TSI (ms)) + (0.0132. plasma creatinine (μmol/l)).

**Table 2:** Final model from bootstrapped multivariable Cox proportional hazard analyses of predictors of mortality

	β coefficient (95% CI)	HR (95% CI)	Z-score	p Value
Posterolateral scar location	2.50 (1.60 to 3.40)	12.2 (4.97 to 30.1)	5.46	<0.0001
CMR-TSI (ms)*	0.01 (0.00 to 0.02)	1.01 (1.00 to 1.02)	3.26	0.0011
Creatinine (μmol/l)	0.01 (0.00 to 0.02)	1.01 (1.00 to 1.02)	2.83	0.0046
Model LR χ^2^: 73.4, p<0.0001				

*Consequently a CMR-TSI of 100 ms is associated with a hazard ratio of 2.

CI, confidence interval; CMR-TSI, cardiovascular magnetic resonance tissue synchronisation index; HR, hazard ratio.

**Figure 1:**
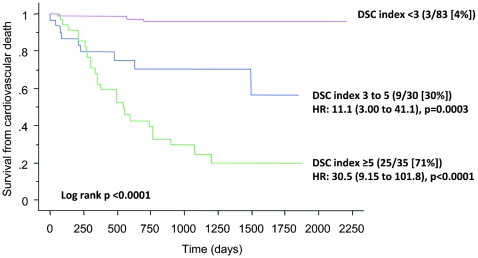
Kaplan-Meier estimates of the time to cardiovascular death. Patients were stratified according to pre-implant DSC index. The event rate, number of patients in the DSC risk stratum and the percentage event rate are shown in brackets. The hazard ratio (HR) and 95% confidence intervals are also shown.

**Figure 2:**
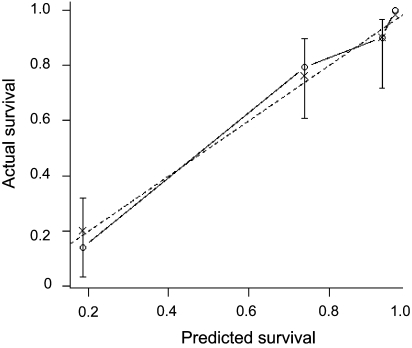
Calibration of predictions of mortality after cardiac resynchronisation therapy. Graph shows actual versus bootstrap-corrected predicted survival.

**Figure 3:**
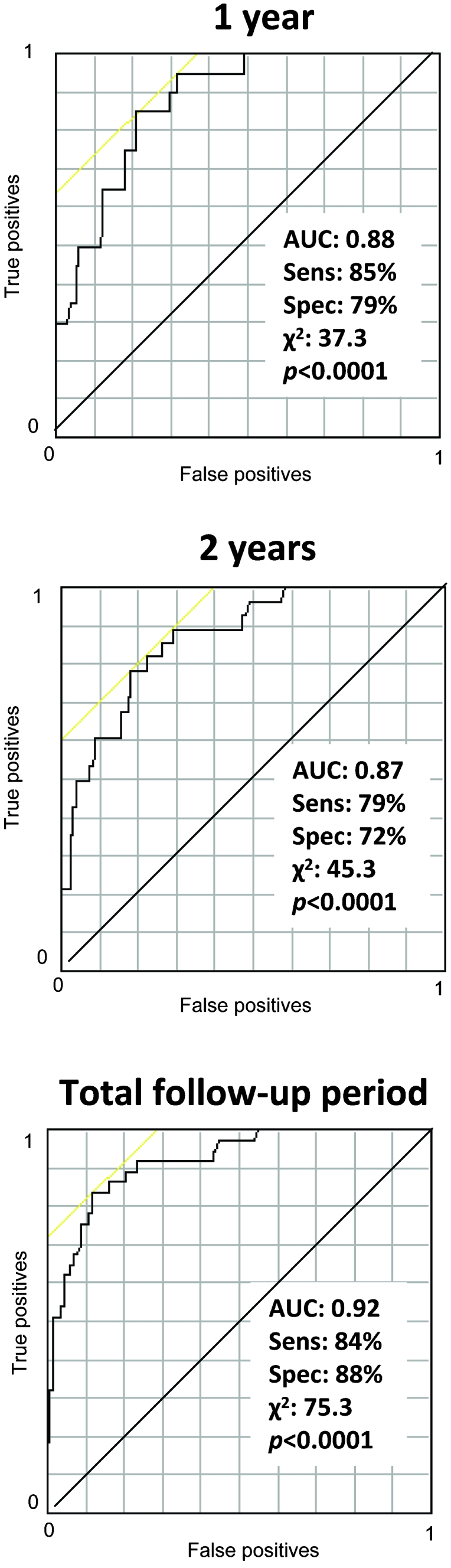
Receiver-operator characteristic curves of the DSC index against cardiovascular mortality at 1 and 2 years, and at the end of the follow-up period. AUC, area under the curve; sens, sensitivity, spec, specificity. Values refer to a DSC cut-off of 4.7.

Strata of the DSC index were designated as low, intermediate or high risk, with cut-offs of <3, between 3 and 5 and ⩾5. These were chosen arbitrarily for ease of use in clinical practice. Kaplan-Meier analyses were used to assess survival according strata of the DSC index (fig 1). Among the statistics derived from Harrell’s routine^18^ ^20^ was a bias-corrected slope of the model of 0.93, indicating an excellent model calibration, taking into account overfitting (fig 2). Somer’s D_app_was −0.70 and the optimism (O) was −0.01. The bias-corrected performance of the stepwise model (D_app_ – O) was acceptable, at −0.69, indicating excellent internal validity. The optimism-corrected discrimination index was 0.91; the optimism-corrected unreliability index U was 0.01 and the overall quality index was 0.85. The ROC for the DSC index in relation to cardiovascular mortality at various time points is shown in figure 3.

### Univariate analyses

Table 3 shows univariate model outputs for other candidate predictors, ranked according to the likelihood ratio (LR) χ^2^. Among the imaging variables, posterolateral scar location (LR χ^2^: 58.6) and CMR-TSI (LR χ^2^: 25.5) emerged as the most significant predictors (all p<0.0001). Scar burden (LR χ^2^: 13.3) and CMR-LVEF (LRχ^2^: 11.1) were also strong predictors (both p<0.001). Among circulating markers, sodium and creatinine were significant predictors (both p<0.01), uric acid only reached borderline significance (p = 0.0808) and haemoglobin failed to reach significance. Male gender was also a modest predictor (p = 0.0417). Age, NYHA class, QRS duration, atrial fibrillation, diabetes or CABG failed to reach statistical significance. Similarly, use of loop diuretics, angiotensin-converting enzyme inhibitors (ACE)/angiotensin-receptor blockers, β-blockers or spirinolactone failed to emerge as predictors of cardiovascular mortality (data not shown). Although ischaemic aetiology did emerge as a significant predictor of cardiovascular mortality in univariate analysis, addition of scar burden to this model rendered aetiology non-significant (data not shown). The decision not to include ischaemic aetiology in the multiple regression model was taken in view of its expected interaction with scar burden and scar location (non-ischaemic cardiomyopathy is associated with little or no scar). In addition, the diagnoses of ischaemic and non-ischaemic cardiomyopathy were made on the basis of size, transmurality and location of scar in the CMR scan.

**Table 3:** Univariate Cox proportional hazard analyses of predictors of mortality

	β coefficient (95% CI)	HR (95% CI)	LR χ^2^	p Value
Posterolateral scar location	2.82 (1.94 to 3.70	16.8 (6.94 to 40.5)	58.6	<0.0001
CMR-TSI (ms)	0.015 (0.01 to 0.02)	1.01 (1.01 to 1.02)	25.5	<0.0001
Scar burden (%)	0.03 (0.01 to 0.04)	1.03 (1.01 to 1.04)	13.3	0.0001
CMR-LVEF (%)	−0.06 (−0.10 to −0.02)	0.94 (0.90 to 0.98)	11.1	0.0032
Sodium (mmol/l)	−0.13 (0.04 to −0.04)	0.88 (0.81 to 0.96)	8.2	0.0031
Creatinine (μmol/l)	0.01 (0.00 to 0.02)	1.01 (1.00 to 1.02)	7.92	0.0049
Transmurality (transmural)	0.92 (0.26 to 1.58)	2.51 (1.29 to 4.84)	7.64	0.0057
Male gender	0.95 (−0.08 to 1.99)	2.59 (0.92 to 7.32)	4.15	0.0417
Uric acid (μmol/l)	0.00 (−0.00 to 0.00)	1.00 (1.00 to 1.00)	2.33	0.1265
NYHA class	0.54 (−0.17 to 1.21)	1.73 (0.84 to 3.37)	2.24	0.1343
CABG	−0.28 (0.18 to −0.62)	0.75 (0.53 to 1.08)	2.42	0.1196
QRS duration (ms)	0.01 (−0.00 to 0.02)	1.01 (1.00 to 1.02)	1.01	0.2202
Age (years)	0.01 (0.02 to −0.01)	1.01 (0.98 to 1.05)	0.78	0.3765
Atrial fibrillation	0.15 (−0.23 to 0.49)	1.16 (0.80 to 1.63)	0.64	0.4220
Diabetes mellitus	−0.17 (−0.55 to 0.28)	0.84 (0.57 to 1.33)	0.61	0.4324
Haemoglobin (g/dl)	−0.02 (−0.19 to 0.02)	0.98 (0.83 to 1.02)	0.51	0.4763

Variables are listed in order of statistical significance and likelihood ratio χ^2^ (LR χ^2^).

CABG, coronary artery bypass graft; CI, confidence interval; CMR-TSI, cardiovascular magnetic resonance tissue synchronisation index; CMR-LVEF, cardiovascular magnetic resonance left ventricular ejection fraction; HR, hazard ratio; NYHA, New York Heart Association.

## Discussion

Our findings have emerged in the context that there are, hitherto, no means of predicting mortality in patients undergoing CRT. Our aim was to identify an index that could predict cardiovascular mortality after CRT on the basis of pre-implant variables. The DSC index, which combines pre-implant measures of dyssynchrony and location of myocardial scar as well as creatinine, is a novel, powerful predictor of cardiovascular mortality in patients treated with CRT. Compared to patients in the lowest risk stratum, cardiovascular mortality values in the intermediate and high-risk strata of the DSC index were 11.1 and 30.5 times higher by the end of the follow-up period, respectively.

The variables from which the DSC is derived have previously been linked to a poor outcome after CRT. Of particular mechanistic relevance to CRT is scar burden and scar location. This study confirms the findings of previous studies showing that the presence of myocardial scar over the posterolateral LV wall is a powerful predictor of cardiovascular mortality.^8^ The precise pathophysiological mechanism is still unclear, but several factors may contribute to this observation. First, myocardial scars are not readily excitable.^30^ A posterolateral scar reduces the volume of excitable myocardium in the vicinity of the LV pacing stimulus and delays activation of potentially recruitable myocardium, which is crucial for CRT.^31^ Second, a posterolateral scar also reduces the amount of myocardium available for contraction in CRT, thereby leading to suboptimal reverse LV remodelling following CRT.^32^ Third, posterolateral scarring may also preclude proper papillary muscle function, which is essential for competence of the mitral valve.

As in a previous study,^6^ we have shown that dyssynchrony, assessed using CMR-TSI, predicts mortality after CRT.^6^ These findings are in contrast to numerous echocardiographic studies showing that increasing dyssynchrony relates to increasing benefit. In other words, our findings point towards a “too much dyssnchrony is bad” paradigm, whereas echocardiographic studies support an “any degree of dyssynchrony is good” paradigm of CRT. Our finding that “too much dyssynchrony is bad” is, however, consistent with the finding that in non-paced patients with heart failure, dyssynchrony, assessed by tissue Doppler imaging, is a predictor of cardiac decompensation.^21^ A radionuclide radioscintigraphy study of patients with dilated cardiomyopathy has also shown that a dyssynchrony index was an independent predictor of major cardiac events, including cardiovascular death.^22^ Moreover, Bax *et al*^33^ recently found that beyond a limit of dyssynchrony (a septal-to-posterior wall motion delay of 100 ms), CRT does not result in reverse LV remodelling. The “too much dyssynchrony is bad” paradigm might be expected in view that dyssynchrony is inversely related to LVEF,^6^ and positively related to LV volumes^6^ and QRS duration.^6^ ^34^ ^35^ Together, these data suggest that the most damaged and worst functioning left ventricles gain the least benefit from CRT. In other words, that there is a maximum of treatable dysynchrony.

Our findings are in contrast to those of the PROSPECT study, in which echocardiographic measures of dyssynchrony were shown to be of little additive value to QRS duration in predicting functional and LV volumetric changes after CRT.^4^ Prominent among the findings of this study were the very high coefficients of variation for echocardiographic dyssynchrony measures, which were as high as 72%.^4^ Current technology, degree of training standards and analytical methods do not allow incorporation of echocardiographic measures of dyssynchrony in a generalised setting, representing a significant limitation for their widespread use.^36^ In contrast, the intra-observer and inter-observer variabilities for CMR-TSI are as low as 3.01% and 8.84%, respectively,^6^ thereby providing the highest accuracy and lowest intra-observer and inter-observer variabilities for a dyssynchrony measure.

We have also considered other non-invasive variables which have previously been shown to be of prognostic value in patients with heart failure treated^7^ ^8^ and not treated^10^ ^26^ ^27^ with CRT. The emergence of plasma creatinine in the predictive model is not surprising because renal impairment is a predictor of poor outcome in non-paced patients with heart failure.^23^ ^24^ ^25^ ^26^ ^26 37^ We have shown that the inverse relation between plasma creatinine and survival still holds in patients with heart failure undergoing CRT.

### Clinical application

Cardiovascular magnetic resonance imaging is becoming a key investigation in patients with cardiomyopathy, as it provides information on aetiology, ventricular function and prognosis.^13^ ^38^ Myocardial tagging,^39^ ^40^ strain-coding^5^ and velocity-encoding^41^ techniques are all capable of providing dyssynchrony measures. These techniques, however, are highly specialised, are not routinely available in any centre and, more importantly, have not been validated against mortality after CRT. In contrast, the CMR technique that allows derivation of the DSC index involves a routine CMR-LGE scan, which is available in any CMR centre. With the emergence of software packages that allow rapid assessment of myocardial scars and wall motion, the DSC index can be easily quantified.

### Limitations of the study

All patients in this study underwent CRT-P. Therefore, the DSC index may not be generalisable to a population of patients treated with CRT-D. External validation of the DSC index in a cohort of patients undergoing CRT-D would be desirable. As a further limitation, the use of β-blockers and spironolactone were relatively low. While drug therapy failed to emerge as a predictor of cardiovascular mortality, we cannot discount the possibility that the relation between the DSC index and cardiovascular mortality might be influenced by more optimised medical therapy. On the other hand, in prospective randomised controlled trials, absence of β-blocking agents or spironolactone was not associated with a significant survival benefit.^1^

**Figure 4:**
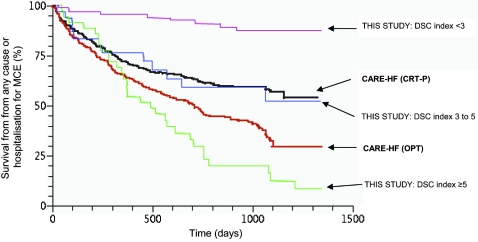
Comparison of events in the present cohort and in the CARE-HF study. Qualitative comparison of Kaplan-Meier survival estimates for the combined endpoint of death from any cause or hospitalisations for major cardiovascular events (MCE) for patients included in this study as well as those in the CRT-P and the optimum medical treatment (OPT) arms the CARE-HF study. Follow-up time in the present cohort has been truncated to 1500 days. Modified with permission from Cleland *et al*.^1^

The DSC index may be useful in predicting the outcome of CRT, but a high DSC index should not be used to deny CRT to patients who satisfy current guideline criteria. As our study did not include a control group, one cannot be certain the CRT-P led to a prognostic benefit. This, however, seems likely in view of the similarity between the prognostic risk profile of the present cohort and that of the CRT-P arm of the CARE-HF study (fig 4).

## Conclusions

This study comprised patients with heart failure in NYHA class III and IV, such as those included in the CARE-HF study. We have found that in such patients, the DSC index is a powerful risk-stratifier for cardiovascular mortality. Patients in the highest risk stratum were more than 30 times as likely to die from cardiovascular causes than those in the low risk stratum. These findings are perhaps not surprising, as extreme dyssynchrony, a posterolateral location of scar and poor renal function have all been linked to a poor outcome in patients with heart failure. The present study supports the use of a standard CMR-LGE in prospective CRT candidates, in whom pre-implantation risk stratification may influence clinical management. Although this study did not include patients undergoing CRT-D, it seems unlikely that patients in the highest DSC risk category, most of whom die of pump failure, would derive a survival benefit from CRT-D. On this basis, the DSC index may influence resource allocation.
